# Ultrasound predictors of peripheral nerve block failure after blast injury: a prospective cohort study

**DOI:** 10.3389/fmed.2026.1826698

**Published:** 2026-07-06

**Authors:** Dmytro Dmytriiev

**Affiliations:** 1 Superhumans War Trauma Center, Lviv, Ukraine; 2 Department of Anesthesiology and Intensive Care, National Pirogov Memorial Medical University, Vinnytsia, Ukraine

**Keywords:** anatomical distortion, blast injury, block failure, hematoma, peripheral nerve block, regional anesthesia, trauma anesthesia, ultrasound-guided nerve block

## Abstract

**Background:**

High-energy blast injuries produce severe anatomical distortion that may compromise ultrasound-guided peripheral nerve blocks (PNBs). However, large-scale data identifying predictors of block failure in this population remain limited.

**Objective:**

To determine the incidence and independent predictors of PNB failure in patients with blast-related limb trauma.

**Methods:**

Prospective observational cohort study conducted between March 2023 and February 2026 at a tertiary war trauma center. Adult patients undergoing ultrasound-guided PNB for blast-related limb injuries were included. Primary block failure was defined as failure to achieve ≥50% reduction in Numeric Rating Scale (NRS) pain score within 30 min together with incomplete sensory blockade in the expected dermatomal distribution. Rescue opioid requirement within 6 h was analyzed separately as a secondary indicator of analgesic burden. Anatomical distortion variables and injury severity were analyzed. Multivariable logistic regression identified independent predictors of failure.

**Results:**

A total of 774 patients were analyzed. Overall block failure rate was 27.8%. Anatomical distortion was present in 64% of cases. Independent predictors of failure included vascular reconstruction (aOR 2.43, 95% CI 1.65–3.58), large hematoma (aOR 1.91, 95% CI 1.43–2.55), scar tissue/adhesions (aOR 1.74, 95% CI 1.29–2.33), fracture displacement (aOR 1.68, 95% CI 1.25–2.24), and Injury Severity Score > 25 (aOR 1.56, 95% CI 1.14–2.13).

**Conclusion:**

Anatomical distortion following blast injury significantly increases the risk of PNB failure. Recognition of trauma-specific anatomical predictors is essential for optimizing regional anesthesia strategies in high-energy injury populations.

## Introduction

**FIGURE 1:**
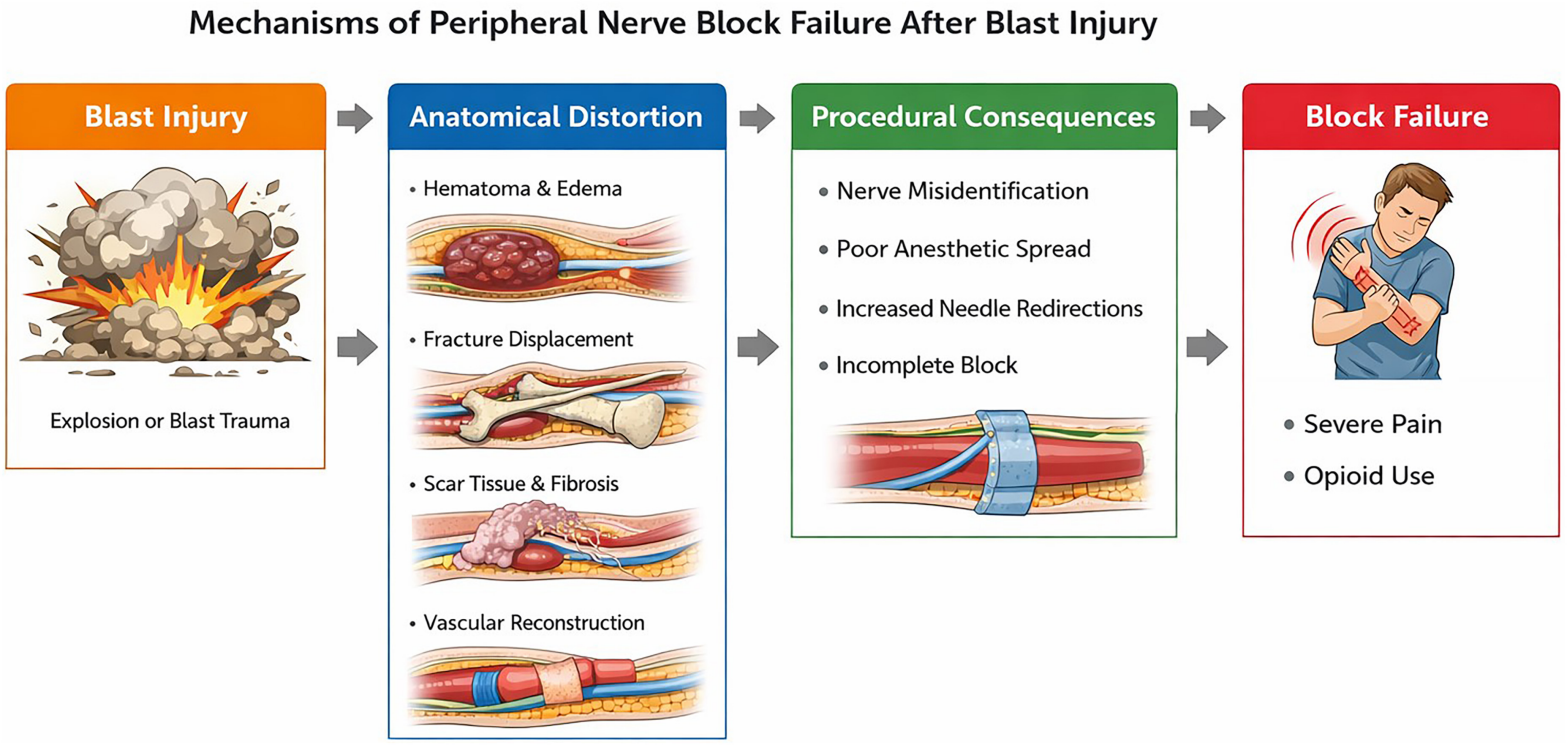
Proposed conceptual pathways linking anatomical distortion and peripheral nerve block failure after blast injury. The figure illustrates proposed conceptual pathways and was not directly evaluated in the present study.

Peripheral nerve blocks (PNBs) represent a cornerstone of multimodal analgesia in patients with severe limb trauma. In combat-related injuries, regional anesthesia is particularly valuable, providing opioid-sparing analgesia, facilitating early mobilization, and potentially influencing the trajectory of chronic pain development (1). In high-energy war trauma, however, the anatomical substrate for safe and effective nerve blockade is frequently profoundly altered. Blast injuries differ fundamentally from civilian trauma in both mechanism and tissue impact (2). The combined effects of overpressure waves, penetrating fragments, secondary tissue displacement, and thermal damage produce complex anatomical distortion (3). Common pathological features include extensive soft-tissue edema, large hematoma formation, disruption of fascial compartments, fracture displacement, vascular reconstruction, fasciotomy wounds, and subsequent scar tissue formation and post-surgical tissue remodeling (4). These alterations may modify the sonographic appearance of neural structures and potentially influence block performance. Ultrasound guidance has become standard practice in regional anesthesia, improving precision and reducing complications compared to landmark-based techniques (5). Nevertheless, most ultrasound-guided PNB techniques assume relatively preserved fascial planes and predictable anatomical relationships. In blast-related trauma, these assumptions frequently fail (2). Nerves may be displaced from expected anatomical compartments, obscured by hematoma, compressed by edema, tethered within scar tissue, or surrounded by vascular grafts (Figure 1) (1). Such distortion may increase technical difficulty, procedural time, needle redirections, and ultimately block failure. Despite the widespread use of ultrasound-guided PNBs in trauma care, systematic large-scale investigations examining predictors of block failure in blast-injured populations remain scarce. Most available literature is derived from civilian trauma settings or small case series, which may not adequately capture the anatomical complexity of high-energy war injuries (5). Understanding the specific anatomical and clinical determinants of PNB failure in blast trauma is essential to improve analgesic strategies, optimize procedural planning, and enhance patient safety (6, 7). The primary objective of this study was to determine the incidence of PNB failure in a large cohort of patients with blast-related limb trauma treated between March 2023 and February 2026. The secondary objective was to identify independent anatomical and injury-related predictors of failure using multivariable analysis. By identifying anatomical and clinical factors associated with unsuccessful blocks, this study aims to inform trauma-adapted regional anesthesia practice in high-energy injury populations.

## Materials and methods

This was a prospective observational cohort study conducted at the Superhumans War Trauma Center, a tertiary referral institution specializing in combat-related injuries. Consecutive adult patients undergoing ultrasound-guided peripheral nerve blocks (PNBs) for blast-related limb trauma between March 1, 2023 and February 28, 2026 were included. The study followed the STROBE recommendations for observational studies. The study protocol was approved by the institutional ethics committee. Written informed consent was obtained from all participants prior to inclusion.

Adult patients aged 18 years or older presenting with blast-related limb trauma, including explosive and drone-associated injuries, who required ultrasound-guided peripheral nerve block (PNB) for analgesia were eligible for inclusion. Only patients undergoing PNB as part of routine clinical trauma care were considered. Enrollment was consecutive during the study period to minimize selection bias.

Patients were excluded if they had documented pre-existing peripheral neuropathy affecting the target limb, coagulopathy or other contraindications to regional anesthesia, or incomplete procedural or follow-up outcome data preventing assessment of block efficacy. A total of 774 consecutive patients met the eligibility criteria and were included in the final analysis. Inclusion Criteria: Patients were eligible for inclusion if they were aged 18 years or older, sustained blast-related limb trauma (including explosive or drone-associated injuries), and underwent ultrasound-guided peripheral nerve block for analgesia as part of routine trauma care during the study period (March 2023 to February 2026). Consecutive enrollment was applied to minimize selection bias and ensure comprehensive capture of all eligible cases. Permission to conduct this observational study was granted by the Institutional Review Board at Vinnytsia National Pirogov Medical University (**IRB_LEC_*N*o7\13072023**), it was registered as a database designed to collect data on war-related chronic pain conditions with the central government committee (*N*o0122U002551)^1^.

Exclusion Criteria: Patients were excluded if they had documented pre-existing peripheral neuropathy affecting the target limb, clinically significant coagulopathy or other contraindications to regional anesthesia, or incomplete procedural or follow-up data that precluded assessment of block effectiveness. Cases in which outcome assessment within the predefined 6-h observation window was not available were also excluded. Clinical, injury-related, and procedural data were prospectively recorded in a structured trauma anesthesia registry throughout the study period. Data collection was performed by attending anesthesiologists immediately after each procedure and subsequently verified by an independent research coordinator to ensure completeness and accuracy. Demographic variables included patient age and sex. Injury-related variables comprised mechanism of injury (blast or drone-related explosion), limb involvement (upper, lower, or combined), presence of open or closed fracture, traumatic amputation, and Injury Severity Score (ISS). Time from injury to peripheral nerve block placement was also recorded. Ultrasound-assessed anatomical distortion variables were documented at the time of scanning prior to needle insertion. Blocks were performed by consultant anesthesiologists or supervised regional anesthesia fellows experienced in ultrasound-guided trauma regional anesthesia. All ultrasound findings were assessed using predefined sonographic criteria agreed upon before study initiation. Formal interobserver agreement testing was not performed. These included the presence of a large hematoma or seroma obscuring fascial planes, fasciotomy or open wound within the scanning field, scar tissue, post-surgical adhesions, or suspected nerve tethering, fracture displacement in proximity to the target nerve, and prior or concurrent vascular reconstruction within the regional anatomical area. Scar tissue and adhesions were defined as hyperechoic disorganized connective tissue, loss of normal fascial plane architecture, tissue remodeling associated with previous surgery, fasciotomy, repeated debridement, reconstructive procedures, or delayed referral after injury. Acute edema alone was not classified as fibrosis. Procedural variables included block type, total procedural time (defined as time from skin preparation to completion of local anesthetic injection), and number of needle redirections. The majority of blocks were performed using ropivacaine 0.5% or bupivacaine 0.25%–0.5% depending on injury location and clinician preference. Typical injection volumes ranged between 20 and 30 mL. Recorded block types included femoral, sciatic, popliteal, brachial plexus, fascia iliaca, and combined regional anesthesia techniques. Perineural adjuncts were not routinely used.

Pain intensity was assessed using the Numeric Rating Scale (NRS) immediately prior to the block and 30 min after completion of injection. Sensory blockade was assessed 30 min after injection using cold sensation and pinprick testing within the expected dermatomal distribution corresponding to the target nerve block. Incomplete sensory loss was considered supportive evidence of incomplete or failed blockade. Pain assessment focused specifically on the injured extremity corresponding to the target peripheral nerve block. Treating anesthesiologists differentiated block-related pain from pain arising from non-blocked injuries based on anatomical localization, dermatomal distribution, and clinical examination. Patients with severe competing pain sources impairing reliable regional assessment were excluded. All outcome data were collected within the predefined 6-h observation window following block placement.

### Statistical analysis

Continuous variables were assessed for normality using the Shapiro–Wilk test and are presented as mean ± standard deviation (SD) for normally distributed data or median (interquartile range) where appropriate. Categorical variables are reported as absolute frequencies and percentages. Comparisons between patients with successful and failed peripheral nerve blocks were performed using the independent samples Student’s *t*-test for continuous variables and the χ^2^ test or Fisher’s exact test for categorical variables, as appropriate. To identify independent predictors of block failure, multivariable logistic regression analysis was conducted. Variables with *p* < 0.10 in univariate analysis and clinically relevant anatomical factors were entered into the multivariable model. Adjusted odds ratios (aORs) with 95% confidence intervals (CIs) were calculated. Model discrimination was assessed using the area under the receiver operating characteristic curve (AUC). Calibration was evaluated with the Hosmer–Lemeshow goodness-of-fit test. Multicollinearity was assessed using variance inflation factors (VIF), with values >5 considered indicative of significant collinearity. All statistical tests were two-sided, and a *p*-value < 0.05 was considered statistically significant. Injury-to-block interval, block type, operator level, local anesthetic selection, and injection volume were evaluated during model development as potential confounding variables.

## Results

A total of 774 consecutive patients were included in the final analysis. The mean age of the cohort was 34.7 ± 9.8 years, and the majority of patients were male (88%). The mean Injury Severity Score (ISS) was 21.3 ± 8.6, with 34% of patients presenting with ISS > 25, indicating severe trauma. Lower limb injuries were the most frequent (51%), followed by upper limb trauma (39%) and combined limb injuries (10%). Open fractures were present in 61% of cases, and traumatic amputations occurred in 18% of patients. Anatomical distortion detected on pre-procedural ultrasound was common. Large hematoma or seroma obscuring fascial planes was identified in 41% of patients, scar tissue, adhesions, or suspected nerve tethering in 37%, fracture displacement in proximity to the target nerve in 46%, fasciotomy or open wound in the scanning field in 34%, and vascular reconstruction in 14% of cases. Detailed baseline characteristics are summarized in Table 1. Lower extremity blast trauma predominated within the cohort, with lower limb injuries accounting for 51% of cases and open fractures present in 61% of patients. Accordingly, sciatic, femoral, popliteal, and fascia iliaca blocks represented the majority of regional anesthesia procedures, whereas brachial plexus blocks were primarily used for upper extremity trauma. Patients with failed blocks demonstrated significantly higher rates of hematoma formation (59% vs. 34%, *p* < 0.001), fracture displacement (61% vs. 40%, *p* < 0.001), scar tissue/adhesions (54% vs. 31%, *p* < 0.001), and vascular reconstruction (27% vs. 9%, *p* < 0.001) compared with successful blocks. Injury-to-block interval was evaluated during model development but was not independently associated with block failure and therefore was not retained in the final multivariable model.

**TABLE 1:** Baseline characteristics and injury profile (*n* = 774).

Variable	Total (*n* = 774)
Age, years (mean ± SD)	34.7 ± 9.8
Male sex, *n* (%)	681 (88%)
Injury Severity Score (ISS), mean ± SD	21.3 ± 8.6
ISS > 25, *n* (%)	262 (34%)
Time from injury to block (hours), mean ± SD	18.4 ± 9.2
Upper limb trauma, *n* (%)	302 (39%)
Lower limb trauma, *n* (%)	398 (51%)
Combined limb trauma, *n* (%)	74 (10%)
Open fracture, *n* (%)	469 (61%)
Closed fracture, *n* (%)	215 (28%)
Traumatic amputation, *n* (%)	142 (18%)
Large hematoma/seroma, *n* (%)	314 (41%)
Fasciotomy/open wound, *n* (%)	266 (34%)
Scar tissue/adhesions or suspected nerve tethering, *n* (%)	289 (37%)
Fracture displacement, *n* (%)	356 (46%)
Vascular reconstruction, *n* (%)	109 (14%)

Overall peripheral nerve block failure occurred in 215 of 774 patients, corresponding to a failure rate of 27.8% (95% CI 24.7%–31.1%). Successful analgesia was achieved in 559 patients (72.2%). Patients with failed blocks had significantly higher injury severity compared with those with successful blocks (ISS 23.6 ± 9.2 vs. 20.4 ± 8.1; *p* < 0.001). Severe trauma (ISS > 25) was more common in the failure group (51% vs. 27%; *p* < 0.001). Anatomical distortion variables were significantly associated with block failure. Large hematoma was present in 59% of failed blocks compared to 34% of successful blocks (*p* < 0.001). Scar tissue, adhesions, or suspected nerve tethering were observed in 54% versus 31% (*p* < 0.001), and fracture displacement in 61% versus 40% (*p* < 0.001). Vascular reconstruction was markedly more frequent in the failure group (27% vs. 9%; *p* < 0.001). Procedural characteristics also differed significantly. The number of needle redirections was higher in failed blocks (6.2 ± 2.7 vs. 3.1 ± 1.9; *p* < 0.001). Procedural time was longer in the failure group (21.3 ± 6.1 min vs. 15.6 ± 4.8 min; *p* < 0.001). Comparative data are presented in Table 2.

**TABLE 2:** Comparison between successful and failed blocks.

Variable	Success (*n* = 559)	Failure (*n* = 215)	*P*-value
Age (years)	34.2 ± 9.5	36.0 ± 10.4	0.04
ISS	20.4 ± 8.1	23.6 ± 9.2	<0.001
ISS > 25 (%)	27%	51%	<0.001
Hematoma (%)	34%	59%	<0.001
Scar tissue/adhesions (%)	31%	54%	<0.001
Fracture displacement (%)	40%	61%	<0.001
Vascular reconstructio*n* (%)	9%	27%	<0.001
Needle redirections	3.1 ± 1.9	6.2 ± 2.7	<0.001
Procedural time (min)	15.6 ± 4.8	21.3 ± 6.1	<0.001

Multivariable logistic regression identified several independent predictors of block failure. Vascular reconstruction was the strongest independent predictor (adjusted OR 2.43, 95% CI 1.65–3.58; *p* < 0.001). Large hematoma (aOR 1.91, 95% CI 1.43–2.55; *p* < 0.001), scar tissue/adhesions (aOR 1.74, 95% CI 1.29–2.33; *p* = 0.001), and fracture displacement (aOR 1.68, 95% CI 1.25–2.24; *p* = 0.002) were also independently associated with failure. Severe injury (ISS > 25) remained a significant predictor (aOR 1.56, 95% CI 1.14–2.13; *p* = 0.01). The regression model demonstrated good discrimination ability with an AUC of 0.79. Calibration was acceptable (Hosmer–Lemeshow *p* = 0.41), and no significant multicollinearity was detected. Detailed regression results are shown in Table 3.

**TABLE 3:** Multivariable logistic regression analysis for block failure.

Variable	Adjusted OR	95% CI	*P*-value
Vascular reconstruction	2.43	1.65–3.58	<0.001
Large hematoma	1.91	1.43–2.55	<0.001
Scar tissue/adhesions	1.74	1.29–2.33	0.001
Fracture displacement	1.68	1.25–2.24	0.002
ISS > 25	1.56	1.14–2.13	0.01

## Discussion

In this prospective cohort of 774 patients with blast-related limb trauma, we observed a peripheral nerve block (PNB) failure rate of 27.8%, which is higher than typically reported in elective or civilian trauma settings. Our findings demonstrate that several anatomical distortion patterns were independently associated with a higher likelihood of block failure after blast injury. Vascular reconstruction emerged as the strongest independent predictor of failure (6). The presence of grafts or repaired vessels may alter normal neurovascular relationships and increase procedural complexity during ultrasound-guided block placement (8). Similarly, large hematomas and fracture displacement distort fascial compartments and shift neural structures from their expected anatomical positions (9). Scar tissue, adhesions, and post-surgical tissue remodeling may modify local tissue architecture and potentially influence injectate distribution, increasing the risk of incomplete blockade. Importantly, patients with higher Injury Severity Scores were more likely to experience block failure (8, 10). Severe trauma may contribute to increased tissue edema, altered compartment dynamics, and enhanced inflammatory responses, all of which can affect local anesthetic diffusion and nerve responsiveness (11). Although our study did not investigate molecular mechanisms, systemic injury burden appears to interact with local anatomical distortion in determining procedural success (12). Failed blocks were associated with significantly more needle redirections and longer procedural times, suggesting increased technical difficulty in anatomically disrupted tissue (2, 13). These findings suggest that anatomical distortion should be considered during procedural planning in patients with blast-related trauma (14). From a clinical perspective, pre-procedural recognition of anatomical distortion should inform a more cautious and structured approach (15). Mandatory Doppler assessment, proximal nerve tracking, consideration of alternative injection levels, and early planning for multimodal analgesia may improve outcomes in high-risk patients (16). Anticipating potential failure is particularly important in severe trauma, where inadequate early analgesia may contribute to central sensitization and chronic pain development (17). This study has several strengths, including a large prospective cohort and systematic documentation of ultrasound findings in a high-volume war trauma center (18). However, it is limited by its single-center design and the inherent constraints of observational methodology. Long-term pain outcomes were not evaluated and should be addressed in future research (19). In conclusion, peripheral nerve block failure after blast injury is closely linked to identifiable anatomical and injury-related factors. Recognition of trauma-specific distortion patterns may improve procedural planning and enhance analgesic effectiveness in high-energy injury populations. This observational study cannot establish causality between anatomical distortion and block failure. Direct assessment of traumatic nerve injury was not performed, and intrinsic neural damage may have contributed to incomplete analgesic response. Pain assessment in polytrauma patients remains inherently complex despite anatomical localization strategies. Additionally, molecular mechanisms related to tissue edema, inflammation, and altered anesthetic diffusion were not directly evaluated. Several limitations should be acknowledged. First, ultrasound classification of scar tissue, adhesions, and suspected nerve tethering was based on predefined sonographic criteria; however, formal interobserver reliability testing was not performed. Second, the observational design precludes causal inference. Third, residual confounding related to block type, local anesthetic selection, injection volume, and operator experience cannot be excluded. Finally, the study was conducted in a single high-volume war trauma referral center, which may limit generalizability.

## Conclusion

Peripheral nerve block failure after blast injury was associated with several identifiable ultrasound-detected anatomical distortion patterns, including vascular reconstruction, large hematoma, fracture displacement, and scar tissue or adhesions. Recognition of these factors may improve procedural planning and risk stratification. Further studies are needed to investigate the mechanisms underlying these associations. Recognition of trauma-specific anatomical risk factors should inform a modified, structured ultrasound approach to regional anesthesia in high-energy injury populations.

## Data Availability

The original contributions presented in this study are included in this article/supplementary material, further inquiries can be directed to the corresponding author.

## Footnotes

1
https://nddkr.ukrintei.ua/
